# Biopesticide Activity of Guayule Resin

**DOI:** 10.3390/plants11091169

**Published:** 2022-04-26

**Authors:** Guayente Latorre, María Mercedes García-Martínez, María Martín-Bejerano, Luis F. Julio, Amaya Zalacain, María Engracia Carrión, Manuel Carmona

**Affiliations:** 1 Catedra de Química Agrícola, Escuela Técnica Superior de Ingenieros Agrónomos y de Montes, Universidad de Castilla-La Mancha, Campus Universitario s/n, 02071 Albacete, Spain; guayente.latorre@uclm.es (G.L.); mariamercedes.garcia@uclm.es (M.M.G.-M.); amaya.zalacain@uclm.es (A.Z.); 2 Kimitec Biogroup S.L, Maavi Innovation Center, Paraje Cerro Los Lobos s/n Vícar, 04738 Almería, Spain; mariamartin@kimitec.com (M.M.-B.); luisf.julio@kimitec.com (L.F.J.); 3 Food Quality Research Group, Institute for Regional Development (IDR), Universidad de Castilla-La Mancha, 02071 Albacete, Spain; mengracia.carrion@uclm.es

**Keywords:** biopesticide, guayule resin, *Tetranychus urticae*, *Bemisia tabaci*, *Myzus persicae*, *Frankliniella occidentalis*

## Abstract

The extensive use of synthetic pesticides has created considerable concern for both human health and the environment, which has prompted the search for safer alternatives, such as the resin of guayule (*Parthenium argentatum*). Thus, in the present study, we aimed to test the biopesticide activity of crude guayule resin and three derived fractions and compare them to reference products that act against four of the most economically significant plant pests: *Tetranychus urticae*, *Bemisia tabaci*, *Myzus persicae* and *Frankliniella occidentalis*. None of the guayule products caused plant damage. The crude guayule resin and the hexane and ethyl acetate fractions displayed moderate to high contact mortality against *T. urticae* and *B. tabaci*, as well as moderate to high antifeedant activity against *T. urticae*, *B. tabaci* and *M. persicae*. No significant activity was observed against *F. occidentalis*. A correlation analysis of the activity and fraction composition revealed that guayulins C and D, isoargentatins A and B, argentatins A, B and D and an unknown compound C6 were likely responsible for the contact mortality. By contrast, the antifeedant activity appeared to be caused by guayulins A and B against *T. urticae* and *B. tabaci* and by guayulins C and D and argentatin B against *M. persicae*. The feeding reduction in *F. occidentalis* was associated with an unknown compound C2 and argentatin C. Therefore, guayule appears to be a promising novel biopesticide.

## 1. Introduction

Synthetic pesticides have been instrumental in boosting agricultural production worldwide; however, their overuse and misuse has led to the serious issues of insecticide resistance, environmental pollution [1] and livestock poisoning, along with broad implications for human health [2]. These issues, coupled with consumer demand for more “natural” products and stricter legislation, have placed an emphasis on the use of alternative or complementary approaches to pest management, such as biopesticides. 

Biopesticides are natural materials that are derived from animals, plants and bacteria, as well as certain minerals, which are used for pest control [3]. They are host-specific [4], more readily biodegradable, less likely to contaminate the environment and less toxic to mammals than synthetic pesticides [5]. 

Biopesticide use on a global scale is increasing by almost 10% every year, which is reducing the overreliance on chemical pesticides [4]. However, they currently only make up a small share of the crop protection market with a value of about USD 3 billion worldwide, which accounts for just 5% of the total market. In the United States market, more than 200 products are available, while the European Union market only has 60 analogues, of which the majority are microbial biopesticides that are derived from *Bacillus thuringiensis* [4].

Over the last several years, plant-based extracts and essential oils have emerged as attractive alternatives [4]. The principal botanical pesticides are essential oils that are produced as secondary metabolites by plants [2], which consist of complex mixtures of volatile and lipophilic bioactive compounds that can be classified into low molecular weight phenylpropanoids and terpenoids, monoterpenes and sesquiterpenes [2]. Depending on the physiological characteristics of the insect species, as well as the type of plant, plant extracts and essential oils exhibit a wide range of action against insects: they can act as repellents, attractants or antifeedants and they can also inhibit respiration, hamper the identification of host plants by insects, inhibit oviposition and decrease adult emergence via ovicidal and larvicidal effects [4]. 

With regards to the marketability of essential oils, they in fact represent a market value that is estimated at USD 700 million and a total worldwide production of 45,000 tons [4]. However, only a small number of botanical pesticides have been commercialized so far, for example, pyrethrin, thymol, azadirachtin, eugenol and citronella oil, and others are still pending approval, such as cinnamaldehyde [6]. Guayule (*Parthenium argentatum* Gray) rubber is a promising substitute for the natural rubber from *Hevea brasiliensis* L. Its resin fraction is potentially a commercial source of several compounds, including biopesticides. As a by-product of rubber processing, guayule resin is a dark green-brown, highly viscous, sticky, water insoluble and semi-solid liquid at room temperature, with a strong and distinct pine/citrus odor. The resin contains a broad variety of secondary metabolites, including monoterpenes and sesquiterpenes (essential oils), sesquiterpene esters (guayulins), triterpenoids (argentatins), organic acids (such as cinnamic, p-anisic, palmitic, stearic, oleic, linoleic and linolenic acids) and polyphenolics [7]. There is some evidence for the effective use of resin extract against pests, such as termites of the genus *Reticulitermes* [8,9,10] and *Coptotermes* [11], and against agricultural pests, such as *Helicoverpa zea* and *Spodoptera exigua* [12]. Therefore, guayule could have potential as a biopesticide against some of the most economically significant pests for fruit, vegetables and ornamental plants worldwide, which are included in the CABI Invasive Species Compendium (ISC) (https://www.cabi.org/ISC, accessed on 17 December 2021) and are described below.

*Tetranychus urticae* (Koch, 1836), also known as the red spider mite, has been reported to attack more than 1200 species of plant and is the most polyphagous of the Tetranychidae family [13]. The red spider mite can cause plant damage directly, through chlorophyll depletion, webbing, defoliation and necrosis in young leaves and stems, or indirectly, by blocking photosynthesis and transpiration, which ultimately kills the plant [14]. *T. urticae* has been reported to develop cross-resistance to conventional insecticides [15] and more than 500 cases of acaricide resistance have been reported for this species according to the Arthropod Resistance Database, which is >90% of all spider mite resistance [16].

The sweet potato whitefly *Bemisia tabaci* (Gennadius, 1889) is one of the most significant agricultural pests in the world. This species attacks a broad range of plants (around 600 different plant species), including horticultural, vegetable, ornamental and industrial crops [17]. Whitefly causes direct damage by ingesting plant phloem and producing honeydew on the surface of leaves and stems, on which sooty mold can grow. Whitefly also causes indirect damage through the transmission of more than 100 different plant viruses, such as begomoviruses (Geminiviridae) [17]. *B. tabaci* has 11 genetic groups that are composed of at least 34 morphologically indistinguishable species [18]. This variability, combined with its capacity to develop cross-resistance to conventional insecticides, makes the control of this insect challenging; so, novel active ingredients with combined modes of action are needed for resistance management programs in order to control this pest efficiently [19].

The green peach aphid *Myzus persicae* (Sulzer, 1776) is the most important aphid virus vector. It has been shown to transmit over 100 plant viruses in over 30 different families, including many major crops [20]. The extensive use of conventional chemical insecticides has led to the development of multiple forms of resistance to most classes of insecticides [21]. 

The western flower thrip *Frankliniella occidentalis* (Pergande) affects commercial plant production in various ways: directly, by reducing yield and market quality through feeding damage or the transmission of virus pathogens, and indirectly, in cases when the mere presence of thrips on a crop is used as a reason to deny its entry into a profitable market [22]. Indeed, both *B. tabaci* and *F. occidentalis* are included on the EPPO A2 list of pests that are recommended for regulation as quarantine pests [23] and, as such, growers may experience the loss of crops and capital due to quarantine requirements [22]. For these reasons, farmers worldwide have utilized broad-spectrum insecticides to control *F. occidentalis*, which has a knock-on effect on production costs. In addition, the overuse of insecticides has led to resistance to numerous insecticides within populations of *F. occidentalis* and the destabilization of integrated pest management programs for other pests [24].

In the present study, we sought to comparatively evaluate the biopesticide activity of crude guayule resin and three derived fractions on four plant pests that have a high negative impact on the agri-food industry versus standard reference products. Secondly, although it is known that guayule resin is active against several pests, it is not known for sure which compounds are responsible for this activity. Therefore, a correlation between the composition and activity of the resin and each of the fractions was carried out in order to attribute the activity to specific compounds.

## 2. Results 

The analyses in this study were designed chiefly to examine the non-toxic modes of action of the candidate pesticides (settling inhibition, egg laying inhibition and antifeedant activity) and compare them to contact toxicity. Ingestion toxicity and/or neurotoxic effects were not evaluated. 

In parallel to the performance of these tests, we also assessed the health of the host plants and found no phytotoxic signs for any of the guayule products.

### 2.1. Guayule Efficacy against T. urticae

As shown in Table 1, crude guayule resin (1%) and its hexane fraction had strong antifeedant effects on *T. urticae* adults, which were comparable to the KIMITEC (K_Prototypes) products. In addition, the hexane fraction caused strong contact mortality that was comparable to the KIMITEC (K_Prototypes) products and was greater than that of the reference product SPINTOR^®^ (under our assay conditions), which is known to controls caterpillars and thrips with ingestion, contact and translaminar activity. The ethyl acetate fraction showed moderate antifeedant effects but no contact mortality.

### 2.2. Guayule Efficacy against B. tabaci

Table 2 summarizes the effects of the different treatments on *B. tabaci* adults. 

Crude guayule resin (1%) and its hexane fraction caused moderate (60–75%) contact mortality, which was higher than the reference product SPINTOR^®^ but lower than the KIMITEC prototypes. In addition, the crude resin showed a strong (> 75%) antifeedant effect (repellency and oviposition), which was higher than that for SPINTOR^®^. No significant antifeedant effects were observed for either the hexane, ethyl acetate or methanol fractions.

### 2.3. Guayule Efficacy against M. persicae

Table 3 summarizes the effects of the different treatments on *M. persicae* adults. No contact mortality against the aphids was found for any of the guayule samples that were assayed. However, the hexane and ethyl acetate fractions displayed moderate antifeedant activity that was comparable to that of the KIMITEC formulations and was higher than that of the SPINTOR^®^ reference product.

### 2.4. Guayule Efficacy against F. occidentalis

As seen in Table 4, no contact mortality or antifeedant activity against *F. occidentalis* adults was observed for any of the guayule-derived samples and all of them showed significantly lower activity than the KIMITEC K_Prototype formulations and the SPINTOR^®^ reference product.

### 2.5. Characterization of Guayule Extracts 

**Figure 1:**
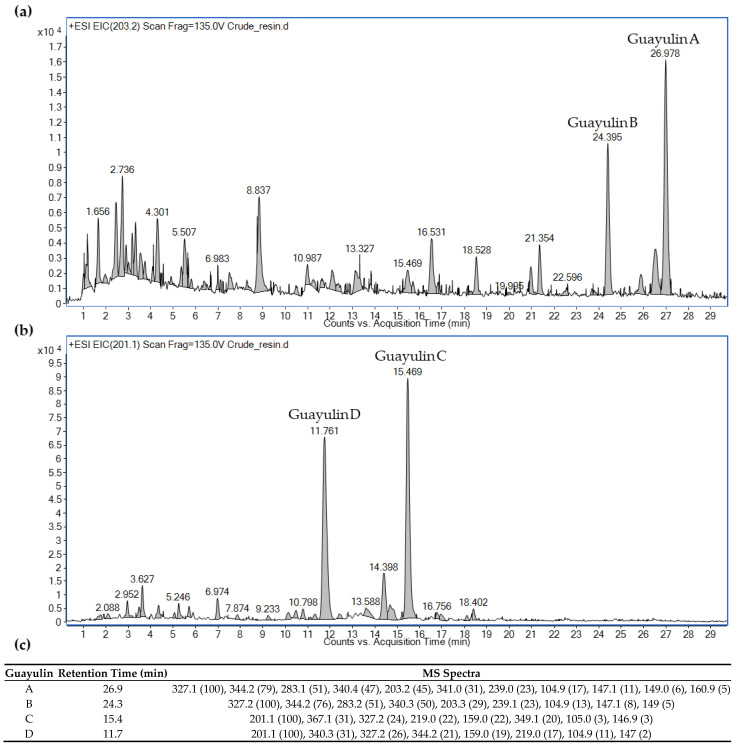
Identification of guayulins in the crude guayule resin based on their mass spectra: (**a**) *m*/*z* 203.2 for guayulins A and B; (**b**) *m*/*z* 201.1 for guayulins C and D; (**c**) retention time and MS spectra of the four guayulins.

The two most abundant families of compounds within guayule resin are the guayulins and the argentatins [25,26]. An analysis of the guayule resin using liquid chromatography–mass spectrometry (see Methods) permitted the identification of four guayulins (A, B, C and D) that was based on their characteristic *m*/*z* values of the product ions (Figure 1). All four guayulins showed characteristic spectral *m*/*z* lines at 105 and 147, which corresponded to C_8_H_9_ and C_11_H_15_, respectively [25]. Guayulins A (retention time (RT), 26.9 min) and B (RT, 24.3 min) had the characteristic *m*/*z* lines at 149, 161 and 203, which corresponded to the bicyclogermacrene structures after fragmentation (Figure 1c) [25]. By contrast, guayulins C (RT, 15.4 min) and D (RT, 11.7) showed the characteristic 147, 159 and 201 *m*/*z* lines, which corresponded to the fragmentation of the aromadendrene structure. In addition, guayulins C and D showed the 219 *m*/*z* spectral line, which corresponded to C_15_H_22_O (Figure 1c) [25]. 

**Figure 2:**
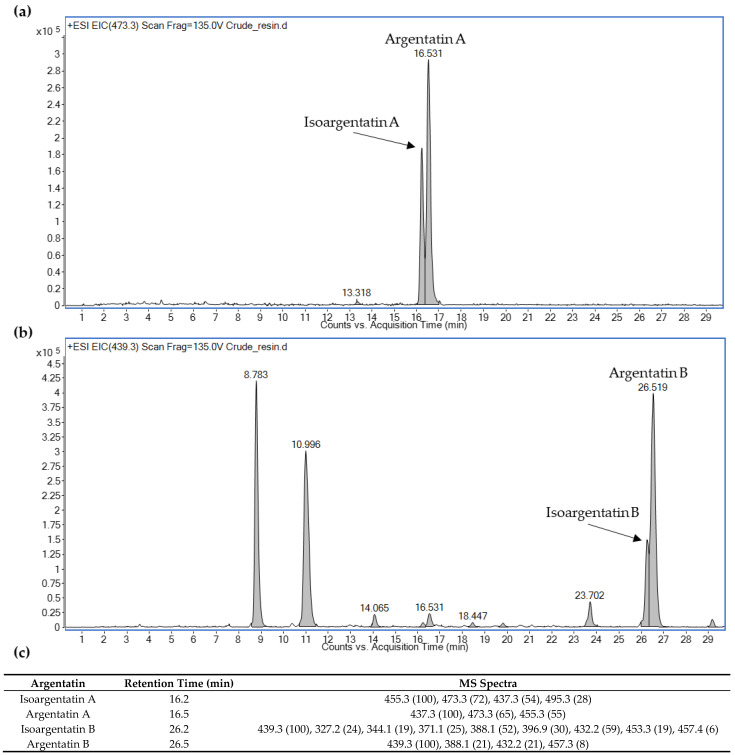
Identification of argentatins and isoargentatins A and B in the crude guayule resin based on their mass spectra: (**a**) *m*/*z* 473.3 for argentatin A and isoargentatin A; (**b**) *m*/*z* 439.3 for argentatin B and isoargentatin B; (**c**) retention time and MS spectra of the four argentatins.

The location of some characteristic *m*/*z* product ions allowed the identification of the two main argentatins (A and B) and their isomers (Figure 2): *m*/*z* 473.3 and 455.3, which corresponded to [M+H]^+^ and [M-H_2_O]^+^, respectively, [25] for argentatin A (retention time (RT), 16.5 min) and isoargentatin A (RT, 16.2 min); and *m*/*z* 457.3 and 439.3, which corresponded to [M+H]^+^ and [M-H_2_O]^+^, respectively, for argentatin B (RT, 26.5 min) and isoargentatin B (RT, 26.2 min).

**Figure 3:**
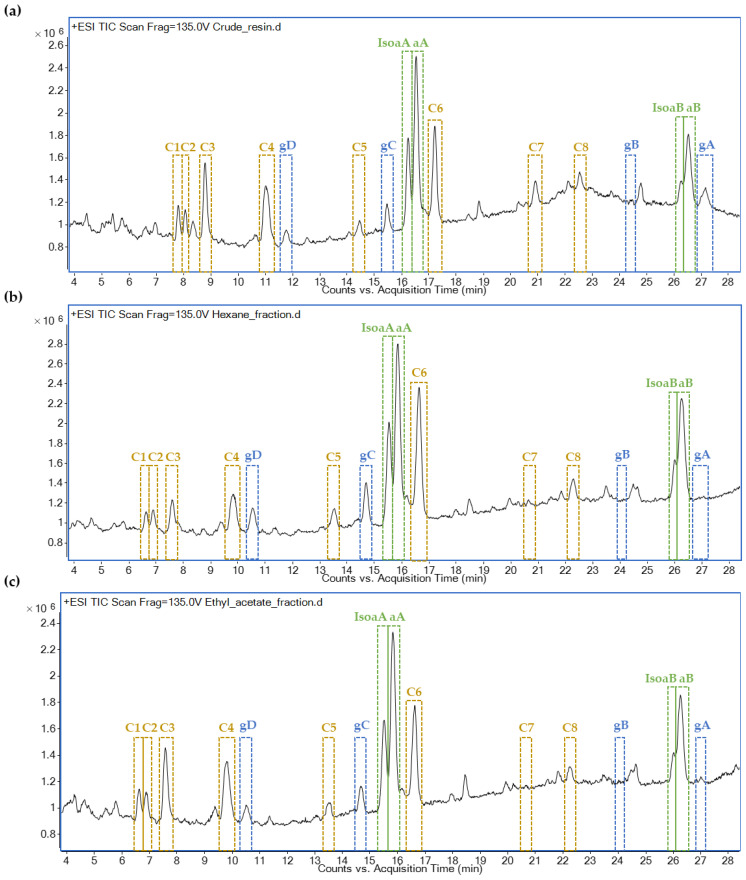
Mass spectrometry total ion chromatogram (TIC) and compound identification of (**a**) the crude guayule resin, (**b**) the hexane fraction and (**c**) the ethyl acetate fraction. Unidentified peaks are highlighted in yellow and named C1–C8, while guayulins (gA–gD) are highlighted in blue and argentatins (IsoaA, aA, IsoaB and aB) are highlighted in green.

The full scan profiles of the three guayule-derived samples (crude, hexane and ethyl acetate fractions) that were obtained by LC–MS were comparable, although the compound areas, and therefore the concentrations, varied between the different samples (Figure 3). In addition to the eight compounds that have already been described (four in each family), another eight compounds, named C1–C8, were evident in relevant amounts. Argentatins A and B and their isomers were the most abundant compounds, but other compounds, including C3, C4 and C6, were also present in significant amounts (Figure 3). 

**Figure 4:**
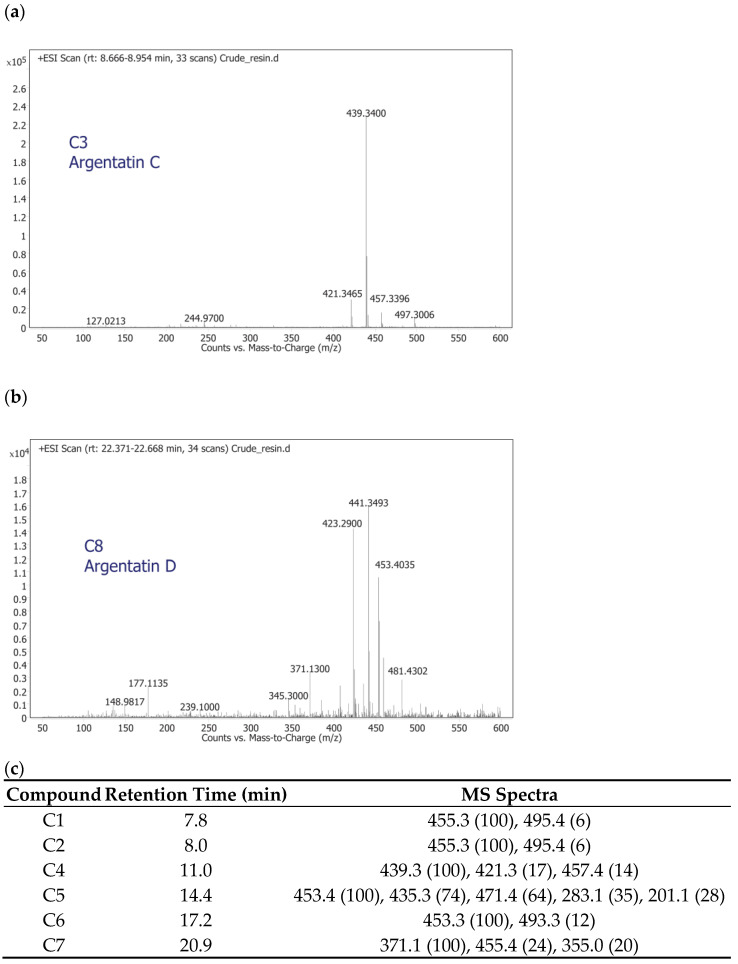
Mass fragmentation patterns in the mass spectrometry total ion chromatogram (TIC) of the tentatively identified peaks for (**a**) C3 (argentatin C) and (**b**) C8 (argentatin D); (**c**) the spectra characterization of the unidentified peaks C1–C7.

Regarding the compounds C1–C8, the presence of common *m*/*z* product ions (Figure 4) suggested that they could all belong to the same family of compounds. In addition, the similarity of the spectra of C1 and C2 and of C3 and C4 suggested that they could be isomers (Figure 4). A literature search allowed us to tentatively identify two of these compounds: C3 and C8 [27]. The C3 spectra (Figure 4a) had two characteristic *m*/*z* lines at 439.3 and 497.3, which corresponded to argentatin C [27,28]. The C8 spectra (Figure 4b) was consistent with the structure of argentatin D, as evidenced by the characteristic 459.3 *m*/*z* line [27,28]. The remaining six compounds could not be identified.

**Figure 5:**
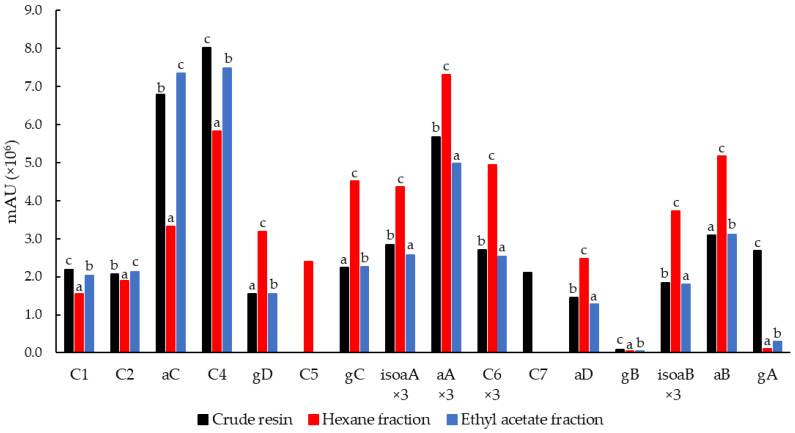
Compound quantification as peak areas for the three guayule-derived samples. For each compound (C1–C7 unidentified compounds; aA–aD, argentatins A–D; isoaA–isoaB, isoargentatins A–B; gA–gD, guayulins A–D), the letters a–c indicate statistically significant differences between the three guayule-derived samples at a 95% confidence level. The peak areas of isoaA, aA, C6, isoaB and aB were divided by a factor of 3 to use the same scale.

All compounds from the full scan were quantified as peak areas (Figure 5) except for guayulin B, which was quantified at *m*/*z* 203.2. The highest proportions of isoargentatins A and B and argentatin A were found in the hexane fraction and the lowest proportions were found in the ethyl acetate fraction. The highest proportion of argentatin B was found in the hexane fraction and the lowest proportion was found in the crude resin. Regarding the guayulins, the polar guayulins C and D were particularly enriched in the hexane fraction, whereas they were the lowest in the crude resin. The apolar guayulins A and B showed the highest concentrations in the crude resin and the lowest concentrations in the hexane fraction. Compounds C1–C8 exhibited very different behaviors across the fractionated guayule samples. Overall, argentatin C, C4 and C6 showed the highest proportions in all samples, whereas C5 was only found in the hexane fraction and C7 was only found in the crude resin. In addition, the concentration of each compound varied significantly between the three guayule samples. The concentrations of compounds C1 and C4 were the lowest in the hexane fraction and the highest in the crude resin. The concentrations of C2 and argentatin C were also the lowest in the hexane fraction but the highest in the ethyl acetate fraction. The concentrations of C6 and argentatin D were the highest in the hexane fraction and the lowest in the ethyl acetate fraction.

To find out which compound(s) was responsible for the activity in each case, the relationship between the activity for each of the insects that were assayed (measured by contact mortality, settling inhibition, egg laying inhibition and feeding reduction) and the concentration of the compound (measured by peak areas) was studied using Pearson's correlation test (Table 5). Compounds C5 and C7 were not considered in this analysis as they were only found in one of the guayule-derived fractions.

For *T.* *urticae* (Table 5), guayulins C and D, isoargentatins A and B, argentatins A, B and D and compound C6 had a strong positive correlation with contact mortality, whereas C1, C2, C4 and argentatin C had a strong negative correlation and guayulins A and B had a moderate negative correlation. However, only the correlations of argentatin A and C2 were significant. With respect to settling inhibition, none of the correlations were significant, but guayulins A and B showed a strong positive correlation, whereas guayulins C and D, isoargentatin B and argentatin B showed no correlation.

Furthermore, argentatin A showed a moderate positive correlation, whereas isoargentatin A, argentatin D, C1, C4 and C6 showed a weak positive correlation and C2 and argentatin C showed a weak negative correlation. Lastly, although none of the correlations were significant, guayulins A and B had a strong direct correlation with egg laying inhibition, whereas guayulins C and D, isoargentatin B, argentatins B and C and compound C6 had no correlation. Isoargentatin A, argentatins A and D and compounds C1 and C4 had a weak positive correlation and argentatin C and C2 had a weak negative correlation. In sum, guayulins C and D, isoargentatins A and B, argentatins B and D, C6 and, especially, argentatin A could cause the contact mortality of *T. urticae*, whereas the settling and egg laying inhibition seemed to be more determined by guayulins A and B.

For *B. tabaci* (Table 5), no compound showed a significant correlation with contact mortality, settling inhibition or egg laying inhibition. Nevertheless, guayulins C and D, isoargentatins A and B, argentatins A, B and D and compound C6 showed a strong positive correlation with contact mortality, whereas guayulins A and B had a weak positive correlation. C1 and C4 had a moderate negative correlation and C2 and argentatin C had a strong negative correlation. As for settling inhibition, guayulins A and B had a strong positive correlation, whereas argentatin A had a moderate positive correlation. Guayulins C and D, isoargentatins A and D, argentatin B, C4 and C6 showed a weak direct correlation, C1 showed no correlation and C2 and argentatin C showed a moderate and weak negative correlation, respectively. Finally, guayulins A and B had a strong positive correlation with egg laying inhibition, whereas guayulins C and D, isoargentatin B, argentatin B and C6 had no correlation. Argentatin A had a moderate positive correlation and isoargentatin A, argentatin D, C1 and C4 showed a weak direct correlation. C2 and argentatin C had a moderate and weak negative correlation, respectively. Thus, polar guayulins C and D, isoargentatins A and B, argentatins A, B and D and compound C6 could cause the contact mortality of *B. tabaci*, whereas apolar guayulins A and B could cause settling and egg laying inhibition, as observed for *T. urticae*.

For *M. persicae* (Table 5), guayulins C and D, isoargentatins A and B, argentatins A, B and D and compound C6 had a strong positive correlation with contact mortality and C1, C2, C4 and argentatin C showed a strong negative correlation. By contrast, guayulins A and B had a moderate negative correlation. Of all the correlations, only those of argentatins C and D and isoargentatin A were significant. With regards to settling inhibition, guayulins A and B and compounds C1 and C4 had a strong negative correlation, whereas guayulins C and D, isoargentatin B and argentatin B showed a strong positive correlation. Additionally, isoargentatin A, argentatin D and C6 had a moderate positive correlation and C2 and argentatin C had a moderate negative correlation. Finally, argentatin A had a weak positive correlation. Thus, guayulins C and D, argentatins A, B and D and compound C6 could be responsible for the contact mortality of *M. persicae* and guayulins C and D, isoargentatin B and argentatin B could be responsible for settling inhibition.

Finally, no correlations were found to be significant for *F. occidentalis* (Table 5). Nonetheless, guayulins C and D, isoargentatins A and B, argentatins A, B and D and compound C6 had a strong positive correlation with contact mortality, whereas C1, C2, C4 and argentatin C presented a strong negative correlation. Guayulins A and B had a weak direct correlation and no correlation, respectively. On the other hand, C2 and argentatin C had a strong positive correlation with feeding reduction, whereas isoargentatins A and B, argentatins A, B and D and compound C6 showed a strong negative correlation. Guayulins A–D had a moderate negative correlation with feeding reduction and C1 and C4 had a moderate and weak positive correlation, respectively. In sum, contact mortality seemed to be linked to polar guayulins C and D, isoargentatins A and B, argentatins A, B and D and compound C6, whereas feeding reduction could be caused by C2 and argentatin C. 

While most of these correlations were not significant, common trends were observed between the concentration and activity for all of the insects that were tested. Guayulins C and D, argentatins A, B and D and compound C6 could be responsible for the contact mortality of all insects. In addition, isoargentatins A and B also seemed to be linked to contact mortality in *T. urticae*, *B. tabaci* and *F. occidentalis*. By contrast, guayulins A and B could cause settling and egg laying inhibition in *T. urticae* and *B. tabaci*. However, *M. persicae* exhibited a slightly different behavior, as settling inhibition appeared to be more related to guayulins C and D, isoargentatin B and argentatin B. Finally, C2 and argentatin C could cause feeding reduction in *F. occidentalis*.

## 3. Discussion

The resin from the guayule shrub contains a variety of secondary metabolites, including terpenoids [7], which are believed to be one of the major chemical categories in plant protection systems against insects [2,29,30]. This makes guayule resin a good candidate for a natural biopesticide. While it is not known precisely which compound(s) is responsible for its insecticide activity [31], the use of guayule resin as a biopesticide has been tested in several studies [8,9,10,11,12], which has paved the way for the evaluation of its efficacy against other insects that have a negative impact on the agri-food industry. To our knowledge, this is the first study to evaluate the activity of guayule resin and other derived compounds against some of the most economically significant plant pests: *B. tabaci*, *T. urticae, M. persicae* and *F. occidentalis*. A previous study tested several compounds that were isolated from guayule resin (partheniol, eudesmol, guayulone, argentone, incanilin, guayulin B and argentatin B) against *M. persicae* [32], of which only partheniol and eudesmol showed antifeedant activity.

Here, protocols were developed to differentiate between the different modes and mechanisms of action. Crude guayule resin and three products that were derived by fractionation with solvents of increasing polarity were tested under laboratory conditions using in vitro (plastic cages) and in vivo (whole plant) models, according to Kimitec protocols for contact mortality and antifeedant activity, as the first step in identifying and enriching the potential biopesticidal compounds within the resin. The concept of the use of an antifeedant as a substance that acts on insect behavior by deterring feeding, settling and oviposition was applied [33]. In some cases, settling inhibition was considered as an indirect repellent effect [34]. Guayule activity was assessed in comparison to two reference formulations that are in the final stage of development for the control of whitefly and spider mites (K_Prototype 3 and 4, respectively), two additional prototypes of broad-spectrum biopesticides that are currently under field validation (K_Prototype 1 and 2) and a commercial natural insecticide (SPINTOR^®^ 480 SC) that is active against a broad range of insect pests [35] and contains the active agent Spinosad, which is a mixture of spinosyns A and B [1].

Both the contact mortality and antifeedant activity that was caused by each fraction varied between insects. Against *T. urticae*, both the crude resin and the hexane fraction had strong antifeedant effects that were comparable to the KIMITEC products and were much higher than the SPINTOR^®^ product. By contrast, the ethyl acetate fraction only showed moderate activity. On the contrary, only the hexane fraction exhibited strong contact mortality, which was comparable to the KIMITEC products but higher than SPINTOR^®^. The strong antifeedant activity of the crude guayule resin against *B. tabaci* was comparable to most of the KIMITEC formulations and was higher than the SPINTOR^®^ reference product, but no antifeedant effects were observed for any of the other fractions. Regarding contact mortality, the crude resin and the hexane fraction showed moderate contact mortality, which was greater than SPINTOR^®^ but lower than the KIMITEC solutions. Against *M. persicae*, only the hexane and ethyl acetate fractions had moderate antifeedant activity, which were again comparable to KIMITEC solutions and higher than SPINTOR^®^. However, no contact mortality was evident for any of the guayule samples. Finally, no activity, neither antifeedant nor contact mortality, was observed against *F. occidentalis.*

In summary, crude guayule resin and/or two of its fractions (hexane and ethyl acetate) had moderate to high antifeedant activity against *T. urticae*, *B. tabaci* and *M. persicae* but no activity against *F. occidentalis*. For contact mortality, the guayule-derived samples performed better than SPINTOR^®^ but worse than the KIMITEC solutions. As with the antifeedant activity, the guayule-derived products performed the best against *T. urticae*. The guayule methanol fraction had no significant activity against any of the tested insects. Overall, the guayule-derived products showed a higher feeding deterrence for all three insects than the commercial SPINTOR^®^ reference product and their performance was similar to that of the KIMITEC products. Additionally, the guayule-derived products performed the best against *T. urticae*.

We used LC–MS to examine the guayule products that were derived from solvent fractionation. In addition to the four guayulins and six argentatins, the peak areas for six other unknown compounds were quantified. The analysis revealed that the profiles of the three guayule-derived samples (crude resin, hexane fraction and ethyl acetate fraction) were similar, as all three essentially contained the same compounds (except for C5, which was only in the hexane fraction, and C7, which was only in the crude resin) although in significantly different concentrations, which could explain the differences that were found in the activity. The crude resin was richer in the apolar guayulins A and B and the compounds C1 and C4, whereas the hexane fraction was richer in the polar guayulins C and D, isoargentatins A and B, argentatins A, B and D and compound C6 and the ethyl acetate fraction was richer in C2 and argentatin C.

The correlation analysis indicated that *T. urticae*, *B. tabaci*, *M. persicae* and *F. occidentalis* could have contact mortality sensitivity to guayulins C and D, argentatins A, B and D and compound C6. *T. urticae*, *B. tabaci* and *F. occidentalis* also showed contact mortality sensitivity to isoargentatins A and B. These compounds were especially enriched in the hexane fraction, which was the fraction that demonstrated the strongest contact mortality activity. 

Antifeedant activity varied among insects. Antifeedant activity was measured indirectly by the inhibition of settling and oviposition in *T. urticae* and *B. tabaci*, both of which were sensitive to guayulins A and B. In the case of *M. persicae*, only settling inhibition was considered, which was found to be affected by guayulins C and D, isoargentatin B and argentatin B. This contrasts with the findings of Bailen et al. (2020) [32], who found that argentatin B and guayulin B had no antifeedant effects on *M. persicae*. However, it should be noted that the testing method was different (they tested direct antifeedant activity, whereas we inferred antifeedant activity as settling inhibition). Moreover, they tested isolated compounds, whereas we used complex fractions. Therefore, it could be that synergies occur in the resin between several compounds that enhances their individual activities. Finally, antifeedant activity was measured directly in *F. occidentalis* by feeding reduction, with C2 and argentatin C being shown as the potential causative candidates. Thus, contact mortality and antifeedant activity in *T. urticae*, *B. tabaci* and *F. occidentalis* could be caused by different compounds, but the two behavioral changes could be caused by the same compounds in *M. persicae*. 

Overall, our findings suggested that guayule-derived products would be good biopesticide candidates for the control of *B. tabaci*, *T. urticae* and, to a lesser extent, *M. persicae*. Moreover, the biopesticide effects seem to be more due to feeding deterrence (settling and oviposition inhibition) than toxic effects, which could be considered as a strength in comparison to conventional broad-spectrum toxic pesticides that have negative effects on non-target organisms [33]. Nevertheless, future studies will have to determine which compound (or compounds) is the cause of this activity. 

The fact that both guayule and Kimitec products are active against *B. tabaci*, *T. urticae* and *M. persicae* is very advantageous because it has been shown that botanical biopesticides perform better when used in combination [33,36], which mitigates the rapid desensitization of insects to individual feeding deterrents following repeated exposure [33]. Along the same line, the large number of active compounds belonging to the two families (sesquiterpenes and triterpenes) that are present in the resin, is a great advantage [25,27]. Interest in the use of guayule resin as a biopesticide is growing as the resin makes it much more difficult for insects to develop a resistance against a single active compound. Further studies are needed to corroborate the activity that has been shown in the isolated compounds and characterize the synergistic effects in depth.

## 4. Materials and Methods

### 4.1. Crude Resin and Fractionation

The five-year-old guayule accession AZ-5 was from an experimental plot in Santa Cruz de la Zarza (Toledo, Spain), which was planted with a density of 33,333 plants/hectare. The sample collection was carried out on 2nd of June 2020 by cutting 5 cm above the ground and the samples were then packaged into kraft bags. To determine the dry biomass, the whole plants were dried at 60 ºC for 48 hours to achieve a moisture content of 4.5–6% and then the leaves were then manually removed from the stem. The stems were then cut into pellets of about 1 cm in length using a manual cutter and were grounded to a 0.5 mm particle size using a centrifuge grinder (Retsch centrifugal Mill ZM1, Haan, Germany). The dried and ground samples of stems were stored in closed screw-cap glass vessels at 4 °C until analysis.

The resin extraction was performed by mixing a sample of 500 ± 0.01 g of guayule stems with 2500 mL of acetone in a flask. The mixture was then heated using a flask heating mantle (6000 mL flask capacity, model 313-500, JP Selecta, Barcelona, Spain) for 45 minutes until boiling. The extraction was carried out using reflux to prevent solvent loss from evaporation.

Once extracted, the resin was cooled and the mixture was first strained to separate the extract (liquid) from the solid guayule residue, followed by vacuum filtering to remove any rubber or other smaller solid residues that remained after the first step. Finally, the filtrate was decanted overnight to remove any remaining solid residue. Once decanted, the resin was transferred into a pre-weighed flask and solvent evaporation was performed in an evaporator bath (Laborota 4000, Heidolph, Germany) at 210 mbar and 50 °C, which yielded the crude resin. 

The crude resin (batch code GR-0720) was then subjected to ultrasound-assisted extraction for 15 minutes using an increasing polarity gradient: (1) hexane, (2) ethyl acetate and (3) methanol. After filtration, the fractions were concentrated in vacuo. The final ready-to-use solutions were prepared at 1% and contained 1% TWEEN-80^®^ in water. TWEEN-80^®^ (1% in water) was used as a negative control.

### 4.2. Insect Populations and Rearing Conditions

Twenty different populations of adult *B. tabaci*, *T. urticae*, *M. persicae* and *F. occidentalis* were sampled from the same number of greenhouses in Almeria (southeast Spain), which is the largest greenhouse producing area in the world. The insects and mites were collected from the leaves of different crops (tomato, pepper, eggplant and zucchini) using an entomological aspirator and/or a fine brush. The insect/mite pest “pools” were reared on *Phaseolus vulgaris* (bean) except for *M. persicae*, which was grown on *Capsicum annuum* (pepper) plants. All populations were maintained in a “walk-in”-type growth chamber at 25 (± 2) °C, 60% relative humidity (RH) and a photoperiod of 16:8 hours (light:dark) until use.

### 4.3. Test Items and Treatments

The test items that were used in this study are presented in Table 6.

Kimitec products (Kimitec Group, Kimitec Agro, Spain) were included for comparative purposes. K_Prototypes 3 and 4 are formulations that are in the final phase of development for the control of whitefly and spider mites, respectively. K_Prototypes 1 and 2 are prototypes of broad-spectrum biopesticides that are currently in the field validation phase. SPINTOR^®^ 480 SC was purchased commercially (Corteva Agroscience Spain, S.L.U, Madrid, Spain). The other test items were manufactured in the Kimitec facilities. In all cases, foliar application was used.

### 4.4. Bioassays 

#### 4.4.1. Contact Mortality (Leaf Disc Method)

The tests were conducted in plastic net bottles (10 cm height, 5 cm diameter, 100–140 micron mesh) and/or Petri dishes (5 cm diameter, 140 microns mesh). A total of 10 replicates for each treatment (test items and negative control) were used in each experiment, which were temporally replicated three times throughout the week. The numbers of live and dead insects and mite adults were counted 24 h post-treatment. Adult insects (whitefly, aphids, thrips) and mites were considered dead when no movement was apparent after probing with the tip of a fine brush under a stereoscopic magnifying glass. Then, the mortality percentage was calculated for the treatment and control groups. When the mortality in the negative control was > 20%, the test was discarded. When the control mortality was >5% but <20%, the observed mortality (%M) was corrected using Abbot´s formula [37]:

(1) $$\%\mathrm{corrected}\;M=\frac{X-Y}{100-Y}\times 100,$$

where X is the mortality (%) in the treatment and Y is the mortality (%) in the control.

The means of %M were compared using one-way analysis of variance (ANOVA, *p* < 0.05) and the LSD test as a post hoc test to verify any significant differences between single treatments using the Statgraphics Centurion XVII software (www.statgraphics.com, accessed on 17 December 2021). When non-normality was detected in the data, an additional Kruskal–Wallis test was conducted and box and whisker plots (median notch) were used. 

#### 4.4.2. Antifeedant Effects (Feeding Deterrent)

The experiments were conducted in bugdorm^®^-type net cages (60 × 60 × 60 cm, 140 microns mesh). Four similar host plants (at the two-leaf stage) were transferred into the same 14 cm diameter pot. Two of the plants were sprayed with the test items, the others were sprayed with the control vehicle and all were allowed to dry for 30 minutes. Target adults (up to 100) were released at a point that was equidistant from the two plants. A total of five cages for each treatment (four plants in each) and the control were used in each experiment, which were replicated three times on different days. Closed and labeled cages were incubated in a “walk-in”-type growth chamber at 25 (± 2) °C, 60% RH and a photoperiod of 16:8 hours (light:dark). After 24 hours, the number of adults that had settled on the treated and non-treated plants and the number of eggs that had been laid on the upper- and underside of each leaf were recorded. The number of adults and eggs were counted using a stereomicroscope.

The settling inhibition index (%SI) was calculated according to the following formula:

(2) $$\%\mathrm{SI}=\left[1-\left(\frac{T}{C}\right)\right]\times 100,$$

where T is the number of insects that has settled on treated plants and C is the number of insects that had settled on the control plants.

The oviposition inhibition index (%IO) was calculated according to the following formula:

(3) $$\%\mathrm{IO}=\left[1-\left(\frac{T}{C}\right)\right]\times 100,$$

where T is the number of eggs that had been laid on treated plants and C is the number of eggs that had been laid on the control plants.

The means of %SI and %IO were analyzed for significance using the non-parametric Wilcoxon signed-rank test (*p* < 0.05). 

In the case of thrips (*F. occidentalis*), it was very difficult to assess the settling inhibition index due to the high mobility of the insects. For this reason, the feeding reduction index (% FR) was calculated instead, which considered the surface of the leaves that presented silver damage symptoms. After 48 hours of treatment, the control and treated host plant leaves were detached and submitted for image processing (Image J. 1.43, 2010, http://rsb.info.nih.gov/ij/, accessed on 17 December 2021) to assess silver damage.

### 4.5. Chemical Profile Using Hplc-MS/MS

Each guayule fraction was dissolved in acetonitrile at a concentration of 1 mg ml^−1^. Then, a 0.5 mL aliquot was filtered through a 0.22 µm nylon filter. The analysis of the compounds that were present in the guayule extracts was performed using an Agilent 1260 Infinity II quaternary liquid chromatograph (Hewlett Packard, Wilmington, NC, USA) with a multiple wavelength detector and a G6420A triple quad LC–MS detector that was equipped with technology electrospray ion source, which was also from Agilent. The chromatographic separations were performed on an Agilent Poroshell C18 column (150 × 2.1 mm, 2.7 μm particle size). The column temperature was 30 °C and the flow rate was 0.8 mL min^−1^. Eluents A and B were used for gradient elution. Solvent A was acetonitrile and solvent B was water. The following gradients were used: 60% A (0–10 min), 80% A (10–20 min), 80% A (20–25 min), 100% A (25–32 min), 100% A (32–37 min), 60% A (37–42 min) and 60% A (42–45 min.). The MS analyses were conducted in positive ion mode and the operating parameters were optimized as follows: ESI–MS/MS in scan mode (*m*/*z* = 50–600) with a scan time of 500 ms and a fragmentor of 135 V. The gas flow and temperature were established at 12 L min^-1^ and 300 °C, respectively. The pressure of the nebulizer was 50 psi and the capillary voltage was established at 4000 V.

### 4.6. Correlation of Activity and Compounds

The Pearson’s coefficient was calculated to assess the correlations of the compound peak areas using the LC–MS measurements and the activity of the guayule-derived samples in the bioassays. 

## 5. Conclusions

Biopesticides have been attracting a lot of attention because they are safer than synthetic pesticides. However, not many biopesticides are currently marketed commercially, so efforts should be made to discover new active substances to allow for the real application of these compounds in the field.

Based on our results, it can be concluded that guayule resin has great potential as a broad-spectrum biopesticide in the agri-food industry, which could allow the cultivation of this shrub to be exploited. However, in order to commercialize guayule as a biopesticide, further investigations are needed: firstly to determine which compounds or synergies of compounds are responsible for the activity, with the aim of achieving the greatest possible activity at the lowest possible dose; secondly to conduct research into the formulation and delivery methods, such as microencapsulation, which could enhance and lengthen the activity, thereby boosting its application potential in the field; and lastly to assess the efficacy of the use of guayule for precise pest problems within diverse cropping systems.

## Figures and Tables

**Table 1:** Efficacy of test items against *T. urticae*.

Treatment	Dose (cc/L)	Contact Mortality (%M) ^1^	Settling Inhibition (%SI)	Egg Laying Inhibition (%IO)
Crude guayule resin	1% (*w/v*)	26.8 ± 10.4 ^d^	97.1 ± 2.1 *	96.3 ± 4.3 *
Hexane fraction	1% (*w/v*)	83.1 ± 5.9 ^ab^	85.2 ± 2.2 *	81.8 ± 5.3 *
Ethyl acetate fraction	1% (*w/v*)	8.8 ± 3.5 ^e^	70.4 ± 7.2 *	68.6 ± 10.1 *
Methanol fraction	1% (*w/v*)	53.1 ± 19.4 ^c^	28.5 ± 10.2	51.4 ± 8.5
SPINTOR^®^ 480 SC (Spinosad)	0.7	21.5 ± 2.0 ^d^	0.0 ± 0.0	45.1 ± 3.3
K_Prototype-1	4	100.0 ± 0.0 ^a^	77.3 ± 6.5 *	78.4 ± 5.3 *
K_Prototype-2	4	100.0 ± 0.0 ^a^	91.4 ± 2.3 *	93.6 ± 2.1 *
K_Prototype-3	4	76.3 ± 8.4 ^b^	92.1 ± 3.3 *	91.9 ± 4.1 *
K_Prototype-4	4	92.6 ± 4.0 ^ab^	98.2 ± 1.7 *	99.0 ± 0.8 *

^1^ %M, % of mortality when Abbot corrected, means within the same column that are followed by the same letter are not significantly different (ANOVA, Kruskal–Wallis, *p* < 0.05); %SI, settling inhibition index; %IO, oviposition inhibition index; * *p* < 0.05, Wilcoxon test.

**Table 2:** Efficacy of test items against *B. tabaci*.

Treatment	Dose (cc/L)	Contact Mortality (%M) ^1^	Settling Inhibition (%SI)	Egg Laying Inhibition (%IO)
Crude guayule resin	1% (*w/v*)	64.8 ± 11.5 ^c^	77.5 ± 6.4 *	86.3 ± 2.7 *
Hexane fraction	1% (*w/v*)	70.7 ± 7.5 ^b^	47.7 ± 9.1	45.4 ± 8.3
Ethyl acetate fraction	1% (*w/v*)	43.5 ± 8.7 ^d^	0.0 ± 0.0	0.0 ± 0.0
Methanol fraction	1% (*w/v*)	38.7 ± 7.6 ^d^	36.8 ± 9.5	45.9 ± 7.7
SPINTOR^®^ 480 SC (Spinosad)	0.7	30.8 ± 3.3 ^e^	0.0 ± 0.0	60.5 ± 7.2 *
K_Prototype-1	4	100.0 ± 0.0 ^a^	77.4 ± 4.2 *	85.2 ± 3.3 *
K_Prototype-2	4	100.0 ± 0.0 ^a^	89.3 ± 4.6 *	98.6 ± 1.1 *
K_Prototype-3	4	98.7 ± 0.9 ^a^	93.9 ± 2.1 *	94.5 ± 3.1 *
K_Prototype-4	4	98.3 ± 1.7 ^a^	30.4 ± 6.2	68.3 ± 5.2 *

^1^ %M, % of mortality when Abbot corrected, means within the same column that are followed by the same letter are not significantly different (ANOVA, Kruskal–Wallis, *p* < 0.05); %SI, settling inhibition index; %IO, oviposition inhibition index; * *p* < 0.05, Wilcoxon test.

**Table 3:** Efficacy of test items against *M. persicae*.

Treatment	Dose (cc/L)	Contact Mortality (%M)	Settling Inhibition (%SI)
Crude guayule resin	1% (*w/v*)	14.8 ± 7.5 ^c^	15.8 ± 7.9
Hexane fraction	1% (*w/v*)	49.1 ± 10.5 ^b^	72.7 ± 6.6 *
Ethyl acetate fraction	1% (*w/v*)	8.8. ± 3.5 ^c^	70.4 ± 5.6 *
Methanol fraction	1% (*w/v*)	53.1 ± 19.5 ^b^	51.4 ± 6.6
SPINTOR^®^ 480 SC (Spinosad)	0.7	24.5 ± 6.4 ^c^	45.0 ± 8.1
K_Prototype-1	4	100.0 ± 0.0 ^a^	73.2 ± 10.2 *
K_Prototype-2	4	100.0 ± 0.0 ^a^	67.7 ± 11.1 *
K_Prototype-3	4	88.1 ± 4.8 ^a^	62.1 ± 9.8 *
K_Prototype-4	4	97.9 ± 2.0 ^a^	61.4 ± 8.9 *

* %M, % of mortality when Abbot corrected, means within the same column that are followed by the same letter are not significantly different (ANOVA, Kruskal–Wallis, *p* < 0.05); %SI, settling inhibition index; %IO, oviposition inhibition index; * *p* < 0.05, Wilcoxon test.

**Table 4:** Efficacy of test items against *F. occidentalis*.

Treatment	Dose (cc/L)	Contact Mortality (%M) ^1^	Settling Inhibition (%SI)	Feeding Reduction (%FR)
Crude guayule resin	1% (*w/v*)	31.8 ± 10.9 ^c^	0.0 ± 0.0	0.0 ± 0.0
Hexane fraction	1% (*w/v*)	37.1 ± 7.7 ^c^	0.0 ± 0.0	0.0 ± 0.0
Ethyl acetate fraction	1% (*w/v*)	20.6 ± 9.1 ^d^	0.0 ± 0.0	20.5 ± 11.2
Methanol fraction	1% (*w/v*)	7.3 ± 7.2 ^d^	0.0 ± 0.0	0.0 ± 0.0
SPINTOR^®^ 480 SC (Spinosad)	0.7	59.8 ± 7.1 ^b^	51.4 ± 7.3 *	49.3 ± 4.2
K_Prototype-1	4	97.5 ± 2.5 ^a^	62.1 ± 9.3 *	82.1 ± 5.2 *
K_Prototype-2	4	97.8 ± 2.1 ^a^	0.0 ± 0.0	86.3 ± 7.3 *
K_Prototype-3	4	60.8 ± 3.7 ^b^	44.4 ± 10.2	42.67 ± 9.1
K_Prototype-4	4	92.1 ± 4.8 ^a^	0.0 ± 0.0	60.91 ± 6.3 *

^1^ %M, % of mortality when Abbot corrected, means within the same column that are followed by the same letter are not significantly different (ANOVA, Kruskal–Wallis, *p* < 0.05); %SI, settling inhibition index; %IO, oviposition inhibition index; * *p* < 0.05, Wilcoxon test.

**Table 5:** Pearson’s correlation tests between the activity and composition of samples for *T. urticae*, *B. tabaci*, *M. persicae* and *F. occidentalis*.

			%M ^1^	%SI	%IO	%FR	C1	C2	aC	C4	gD	gC	isoaA	aA	C6	aD	gB	isoaB	aB	gA
*T. urticae*	%M	PC	1	0.293	0.206		−0.899	−1.000 **	−0.994	−0.891	0.973	0.970	0.995	0.998 *	0.985	0.994	−0.389	0.976	0.972	−0.353
		Sig.		0.811	0.868		0.288	0.008	0.068	0.300	0.149	0.155	0.062	0.039	0.110	0.067	0.745	0.140	0.151	0.770
	%SI	PC		1	0.996		0.155	−0.281	−0.189	0.173	0.062	0.053	0.198	0.350	0.124	0.190	0.767	0.077	0.060	0.791
		Sig.			0.057		0.901	0.819	0.879	0.889	0.960	0.966	0.873	0.772	0.921	0.878	0.444	0.951	0.962	0.419
	%IO	PC			1		0.243	−0.194	−0.100	0.260	−0.027	−0.036	0.109	0.265	0.035	0.101	0.821	−0.012	−0.030	0.843
		Sig.					0.844	0.876	0.936	0.832	0.983	0.977	0.930	0.829	0.978	0.935	0.387	0.992	0.981	0.362
*B. tabaci*	%M	PC	1	0.826	0.764		−0.491	−0.816	−0.757	−0.475	0.668	0.661	0.763	0.855	0.713	0.758	0.204	0.679	0.666	0.242
		Sig.		0.381	0.446		0.673	0.393	0.454	0.685	0.535	0.540	0.448	0.347	0.495	0.452	0.869	0.525	0.536	0.845
	%SI	PC		1	0.995		0.086	−0.348	−0.257	0.104	0.132	0.123	0.266	0.415	0.193	0.258	0.720	0.147	0.129	0.746
		Sig.			0.065		0.945	0.774	0.835	0.934	0.916	0.922	0.829	0.728	0.876	0.834	0.488	0.906	0.917	0.463
	%IO	PC			1		0.187	−0.250	−0.156	0.205	0.030	0.021	0.166	0.320	0.092	0.158	0.787	0.045	0.027	0.811
		Sig.					0.880	0.839	0.900	0.869	0.981	0.987	0.894	0.793	0.941	0.899	0.423	0.971	0.983	0.398
*M. persicae*	%M	PC	1	0.409			−0.937	−0.996	−1.000 **	−0.931	0.990	0.989	1.000 **	0.988	0.997 *	1.000 **	−0.476	0.992	0.990	−0.441
		Sig.		0.732			0.227	0.053	0.007	0.239	0.088	0.094	0.001	0.100	0.049	0.006	0.684	0.079	0.090	0.709
	%SI	PC		1			−0.702	−0.331	−0.419	−0.714	0.531	0.538	0.410	0.261	0.477	0.417	−0.997 *	0.518	0.533	−0.999 *
		Sig.					0.505	0.786	0.725	0.493	0.644	0.638	0.731	0.832	0.683	0.726	0.048	0.653	0.642	0.023
*F. occiden.*	%M	PC	1			−0.949	−0.585	−0.875	−0.825	−0.571	0.747	0.741	0.831	0.908	0.787	0.826	0.093	0.757	0.745	0.131
		Sig.				0.204	0.602	0.321	0.382	0.613	0.463	0.469	0.376	0.275	0.423	0.381	0.941	0.453	0.465	0.916
	%FR	PC				1	0.301	0.679	0.606	0.283	−0.500	−0.492	−0.613	−0.730	−0.553	−0.607	−0.401	−0.513	−0.497	−0.437
		Sig.					0.806	0.525	0.586	0.817	0.667	0.673	0.580	0.479	0.627	0.585	0.737	0.657	0.669	0.712

^1^ %M, contact mortality; %SI, settling inhibition; %IO, oviposition inhibition; %FR, feeding reduction; C1, C2, C4 and C6, unidentified compounds; aA and aD, argentatins A and D; isoaA and isoaB, isoargentatins A and B; gA–gD, guayulins A–D; PC, Pearson correlation; Sig., bilateral signification; * correlation is significant at the 0.05 level; ** correlation is significant at the 0.01 level.

**Table 6:** Test items.

Name	Dose	Target	Batch code	Type
Crude guayule resin	1% (*w/v*)	Unknown	GR-0720-CR	Candidate
Hexane fraction	1% (*w/v*)	Unknown	GR-0720-H	Candidate
Ethyl acetate fraction	1% (*w/v*)	Unknown	GR-0720-EA	Candidate
Methanol fraction	1% (*w/v*)	Unknown	GR-0720-M	Candidate
SPINTOR^®^ 480 SC	0.7 cc/L	Broad-spectrum	N/A	End-use product (EP)
K_Prototype-1	4 cc/L	Broad-spectrum	K1-2020-1	Prototype
K_Prototype-2	4 cc/L	Broad-spectrum	K2-2020-1	Prototype
K_Prototype-3	4 cc/L	*B. tabaci* (main), side pests	K3-2020-0021	End-use product (EP)
K_Prototype-4	4 cc/L	*T. urticae* (main), side pests	K4-2020-0003	End-use product (EP)

## Data Availability

The data presented in this study are available upon request from the authors.
